# A Framework for Off-Line Operation of Smart and Traditional Devices of IoT Services

**DOI:** 10.3390/s20216012

**Published:** 2020-10-23

**Authors:** Chung-Yen Wu, Kuo-Hsuan Huang

**Affiliations:** Department of Computer Science and Engineering, Tatung University, Taipei 104, Taiwan; d10506001@ms.ttu.edu.tw

**Keywords:** off-line control, MQTT, smart and traditional devices, internet of things

## Abstract

Recently, with the continuous evolution of information technology, various products such as Building Information, Internet of Things (IoT), Big Data, Cloud Computing and Machine Learning have been developed and have created a lifestyle change. A smart Internet of Things (IoT) system is formed by combining the communication capabilities of the internet with control, monitoring and identification services to integrate people, things and objects. However, in some IoT environments that have a weak signal, such as remote areas, warehouses or basements, the network may become unstable, meaning that the IoT system is unable to provide efficient services. This paper therefore presents a framework that ensures the reliability of IoT system services so that even if the IoT system cannot connect to the network, the system can provide the services offline. To avoid increasing the installation cost or replacing existing traditional devices with modern smart devices, this framework can also be used to control traditional devices. The system operation is convenient because users can operate all their smart and traditional devices under the IoT system through voice commands and/or a handheld microcontroller, thus reducing the manual operation of the user. The framework proposed in this paper can be applied to various smart scenarios, including smart warehouses, smart restaurants, smart homes, smart farms and smart factories, to improve people’s quality of life and convenience, and create a humane and comfortable smart living environment.

## 1. Introduction

With the advancements in wireless network technology and the development of the Internet of Things (IoT), many devices can now connect to a network to communicate with each other and provide services. The sensors in the IoT system can be used to integrate smartphones, tablets and microcontrollers and to provide various applications in the home and business fields [1,2,3,4,5]. The device-to-device connection enables automatic control to improve people’s quality of life. With the emergence of Industry 4.0, IoT, big data, cloud computing, machine learning and artificial intelligence can effectively contribute to the industrial field [6,7]. Various devices in the industrial field are connected to the IoT through wireless sensor networks to produce different applications. These applications have significantly improved resource management, personnel monitoring and equipment operation in the industry. According to Statista’s [8] research institutions, there will be 31 billion connected devices by 2020 and 75 billion devices by 2025, which shows that the overall ecological development is rapid. While many IoT service platforms [9,10,11] have been developed to integrate these devices in recent years, most of them are nonstandard service and management architectures, which have potential security issues.

The network is crucial to the effectiveness of an IoT system’s services. If the network cannot be successfully connected, the functions of the IoT device will fail. To ensure the IoT system will operate continuously even in places where the network is unstable (e.g., farms, warehouses, dams and forests), this research introduces a framework for edge computing [12]. However, the huge amount of calculations involved in edge computing cannot be used in IoT devices with a low calculation capacity and storage space and large power consumption. Therefore, in a smart home, smart grid or smart city environment, the local device transmits data to the gateway via wireless communication, i.e., edge computing implemented through the computing capabilities of gateways [13,14,15,16,17,18]. In addition, the introduction of boundary computing into the IoT system increases the reliability of the IoT system.

This paper also introduces voice services to control local devices and increase the convenience and usability of the IoT system, which includes smart and traditional devices. Observing [19,20,21], the use of gesture and voice recognition technologies has increased. The feedback of sound and vision allows users to feel that they are no longer just operating products unilaterally, but that they are also enjoying the products. For visually impaired people [22] or people with reduced mobility [23], voice control can help in the operation of the system. Therefore, in the future, gesture recognition and voice control technologies will be more widely used in humanised interfaces to increase the convenience of the IoT system.

In conclusion, the contributions of this paper are summarised as follows:

(1) We proposed a framework for edge computing to support operations in the IoT environment, including traditional devices and smart devices. Then, we used the data aggregation feature of the gateway to perform edge computing and output the instructions corresponding to the device.
(2) We performed device authentication and authorisation in an offline environment to ensure system security.
(3) We provided a method to synchronise the status of devices in a group to prevent repeated operations of devices, and provided use cases for smart hotels and smart factories.

The remainder of this paper is organised as follows: Section 2 introduces the relevant background knowledge and a review of the related research. Section 3 introduces the modular framework. Section 4 displays the data response time of the system. Section 5 introduces the scenarios and choice of modular frameworks that the framework can apply. Section 6 presents the research results and discusses future research directions.

## 2. Related Work

IoT devices are computing devices that connect wirelessly to the network to interact and exchange data, such as electronic products, software, sensors and actuators; these devices can be embedded into vehicles, industrial machines, agricultural tools and home appliances. Due to the convenience brought by the cloud (i.e., a server that does not require management and maintenance, and has unlimited storage space, security management and device identification), IoT applications are often integrated with cloud services [1,2,3]. However, when these IoT devices lose internet connectivity for some reason, such as information security attack or network disconnect, they cannot access cloud services and, thus, local services cannot be used. Therefore, some IoT application services are moved to the local device so that essential features can still be accessed.

However, most devices in the IoT system are limited in terms of computing resources, memory, data storage space and transfer speed. As the transmission and application are the most critical core values of the IoT system, some services in the cloud are moved to the IoT device to perform essential features; however, this requires a lot of computing capability. For example, moving the recognition model, action model and calculation function into an anti-theft camera that uses artificial intelligence (AI) face recognition will require a huge amount of power consumption. Therefore, a device with higher computing capability is required to assist with computing-related models. As the functions of the IoT system have become increasingly complex, Zhong et al. [24] transferred some of the data processing functions, such as the local sensors, actuators and cloud, to the gateway. Services that handle the communication between the local end and the cloud can complete the monitoring and management of the IoT system and analyse and process the collected and stored data. In an IoT scenario, edge computing provides computational power, secures access to the edge data, reduces latency for time-sensitive applications, supports performance in low-bandwidth environments and eases overall network congestion [25]. In this way, merging multiple end devices into a single module can significantly reduce the calculation amount of the end device, and edge computing can be implemented [6,7,13,14,15,16,17,18].

In past research, edge computing was implemented to ensure the reliability in local, and the communication latency reduced [6,7,13,14,15,16,17,18]. However, in these works of literature, most of them are assisted by an additional device server; this is an additional overhead to the hardware cost and should consider whether it is necessary, for example, Smart Farm, where communication of the network is poor. To ensure the reliability of Pesticide Spray Drone [26] to location, another device server that performs boundary operations is set-up. However, most end devices in Smart Farm are relatively simple (detecting temperature, humidity and brightness; switch on or off the water valves, etc.), i.e., it does not require complex computing. Therefore, this study moves the essential functions of IoT applications to the gateway. Compared with [6,7,13,18] and this study, it is feasible to use the more substantial computing power of the gateway to perform edge computing to assist other end devices to compute. Then, edge devices can not only transmit data but also preprocess data and analyse real-time data, which can support edge computing and provide logic control services.

To bring technology into people’s lives, the original business model has been changed [27,28]. The new business models focus on obtaining relevant usage data, delivering value and generating innovative and differentiated value, and they aim to connect data with physical devices to better control and understand each device. This method allows these devices to share data, improve processes and drive growth. Although the transformation and digitisation of the industrial chain has made the management of the industry more convenient, allowing consumers (users) to experience the convenience and progress brought by technology, most traditional devices are unable to connect to the network via wire or wirelessly for data exchange [29]. However, it is not feasible for businesses to purchase all new devices and equipment for a transformation. Therefore, previous research [2,4,14] has used interfaces to receive instructions or collect user data to overcome the bottlenecks caused by traditional devices. Interfaces can maintain the communication (control) mode between the device and the controller, and the controller can receive control from a network request or instruction to take control of the traditional devices.

The IoT devices communicate in local, and effective messaging protocols include Message Queuing Telemetry Transport (MQTT), Constrained Application Protocol (CoAP), Advanced Message Queuing Protocol (AMQP) and HyperText Transfer Protocol (HTTP). As different messaging protocols have different rates of power consumption, communication distance and security, they are applied in different scenarios. The architecture mentioned in this paper can be used with both smart-device controllers (SDCs) and traditional-device controllers (TDCs), which include infrared emitters (IR-emitters) and relay device controllers. When these multiple devices communicate, they take up some of the network bandwidth, which can be expensive. A comparison of CoAP and MQTT in the existing literature [30] showed that CoAP has better network overhead, scalability and latency. However, considering the maturity, one-to-many communication environment, and under congested or poorly reliable networks, MQTT based on TCP has become a crucial decision factor. This paper uses MQTT for communication between devices, which can perform one-to-many communication. MQTT based on TCP is more reliable than CoAP, and because its data packet transmission is small, and because of the low local power consumption, it is suitable to use.

As the IoT system can be used to control several devices, there are specific requirements for device security management. A forged controller controls the smart devices and then obtains the device’s resources. Thus, it is necessary to verify the control source and control target. Gope et al. [31] introduced a lightweight authentication and authorisation mechanism to protect the local device from physical and cloning attacks and to guarantee the anonymity of the device. Although [31] shows excellent performance and security, for existing traditional devices, there is no network or computing capability, and it is not possible to independently complete authentication and authorisation mechanisms. This paper uses the controller device’s authentication and authorisation mechanism to allow the system to register with the gateway during initialisation. When the smart controller uses MQTT [32] to communicate with traditional controllers, devices that are not registered in the gateway are unable to obtain messages, ensuring minimal security and flexibility.

This article presents a framework that allows the IoT system to include essential features when there is no internet access. This architecture can also perform authentication and authorisation when there is no network, to ensure system security. A voice module is added to this architecture to increase the operability of the entire system.

## 3. Proposed Framework

Based on the needs of the establishment of low-cost IoT services and off-line operations, this study proposes a new IoT framework that can realise offline operations without the need to replace existing equipment. Digital transformation is suitable for traditional industries in various industries.

**Figure 1:**
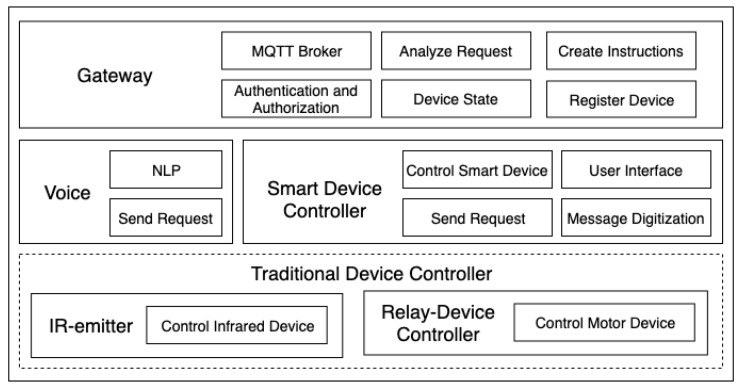
The proposed framework.

**Figure 2:**
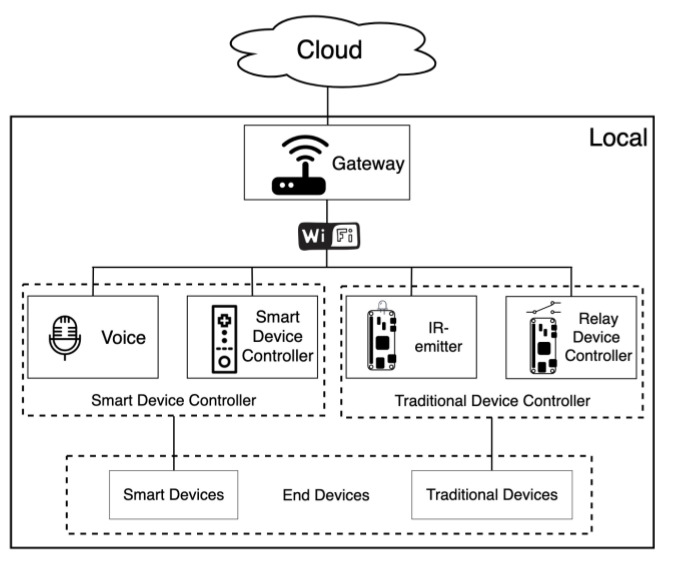
Default system architecture.

Figure 1 shows the proposed modular framework, which can provide a low-cost digital transformation and voice and offline operation of the IoT system. The framework can be divided into five modules: Gateway, Voice, SDC, IR-emitter and Relay-Device Controller. Figure 2 shows the default architecture. The end devices in Figure 2 are controlled by the SDC and the TDC, and the instructions of the controller are parsed by the gateway and passed through MQTT transmission. This architecture simplifies the system: When a new end device is added, only the relevant control instructions of the device need to be added to the gateway, and each controller does not need to be updated individually, thus significantly reducing the complexity of the system update.

In this framework, to prevent unauthorised external devices from efficiently controlling other internal devices, the device controller needs to be authorised via an authentication mechanism. The framework also needs to pay attention to the control of multiple controllers because of the synchronisation between controllers. Therefore, the gateway is responsible for registering the device, performing authentication and authorisation, handling user requests, publishing instructions and determining the device status in the system design.

The following sections describe the functions of each module and the algorithms for identity verification and status synchronisation in more detail. Note that module functions can be swapped according to the user’s needs and the environment. An IoT system that is suitable for each industry has been deployed.

### 3.1. Gateway Module

As a primary communication network for end devices in local and cloud servers, the gateway provides services such as data aggregation, pre-processing, offloading and control feedback. As the gateway has a mechanism for data aggregation, it proceeds with data processing and analysis. As described above, the gateway module performs most of the system functions, including the MQTT broker messages, device registration, authentication and authorisation, and user request analysis. The gateway analyses the user’s request and gives feedback (device instruction) when it is offline or connected to the foreign network. When a gateway has a foreign network, it determines whether the end device needs cloud services, such as AI, sending an email, sending text messages, accessing a database, image analysis and transmitting large-scale data. If necessary, the gateway can request related cloud services for processing. The gateway stores the items that need to be processed by the cloud service when offline and requests the cloud service again when the network recovers.

**Figure 3:**
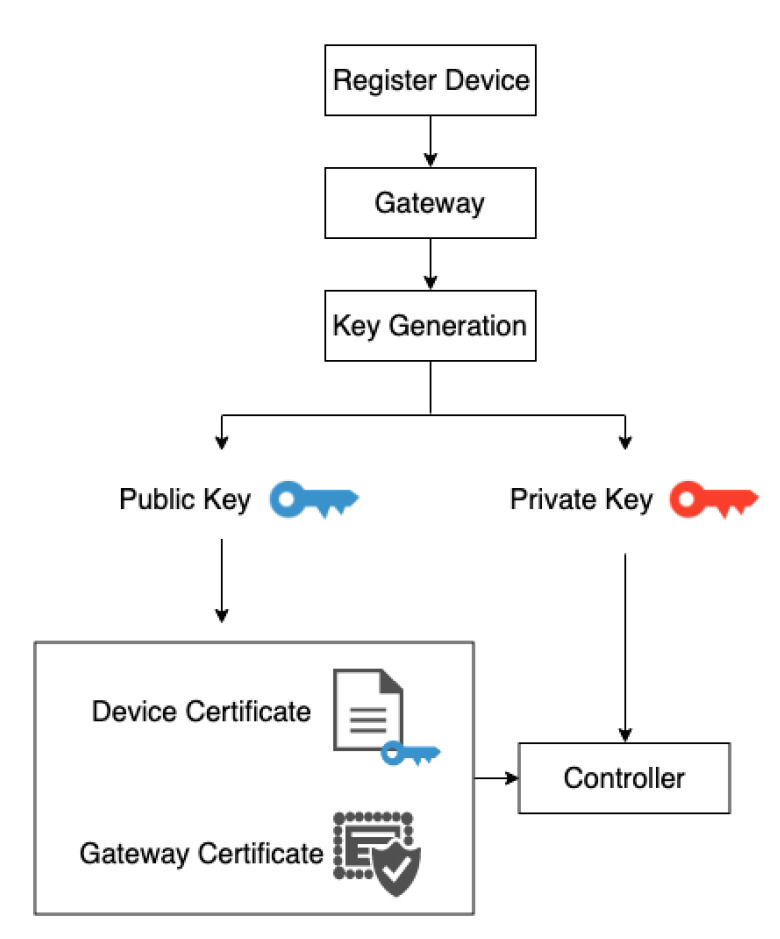
The flowchart of device registration.

**Figure 4:**
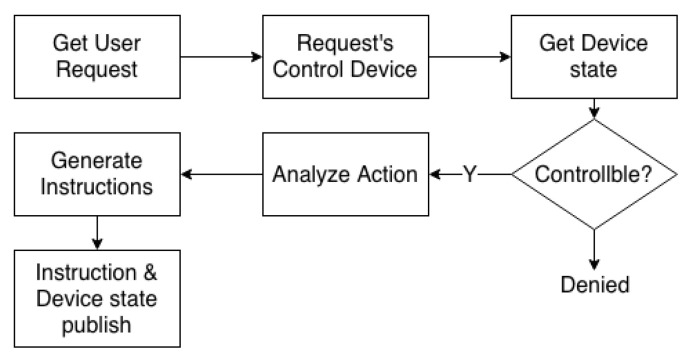
The flowchart of the analysis of user request.

**Figure 5:**
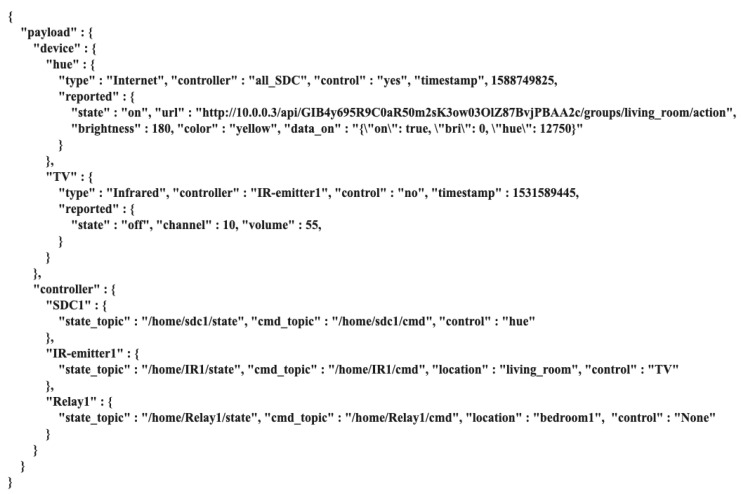
Device status.

This centralised management method is convenient for future system expansion and maintenance and allows the SDC to control the smart device without knowing the device instructions in detail. If not reducing the gateway’s original role that relays internet to the place, another device can be deployed to replace the role of the gateway module. However, the gateway has additional computing power. This research uses the gateway as a centralised component that connects devices, analyses user requests and transmits instructions, reducing the cost of system deployment. The SDC publishes user request messages, including button messages, rotation messages and voice messages. The gateway analyses the message to determine what kind of device instructions the device wants to control, and publishes the instructions. If new devices are added in the future, the user need only update the gateway code, thus reducing the complexity of the system’s update code. The following are the module features of the gateway:

- MQTT Broker: In this module, the MQTT protocol [32] transmits the published messages from the controller to the gateway, and the gateway processes the received messages to determine the corresponding device instructions. The gateway analyses, processes and publishes all messages. Using this method, the system can manage and realise the function of operation centrally and offline. All data and messages are transmitted to the gateway. Thus, if there is a need to connect to an external network, the gateway connects to an external cloud service. Such an architecture can ensure local service reliability, and the system can perform local control even without an external networking function.
- Device Register: This is an essential requirement for the IoT system. To prevent the devices in the system from being controlled by unauthorised controllers and avoid impersonation attacks, authentication and authorisation mechanisms are used. As the instruction judgment and processing of the system are parsed and published by the gateway, each device connected to the gateway needs to register its device identity. Similarly, when the system is initialised, each controller connected to the gateway needs to be registered with the gateway (Figure 3). The gateway generates a 2048-bit RSA key pair and an X.509 certificate using the generated public key. The gateway defines a set of policies to allow or deny access control, such as publishing or subscribing to MQTT or a device state. Then, the device certificate, 2048-bit RSA key pair and gateway certificate as a root Certificate Authority (CA) are stored in the controller for subsequent authentication and authorisation by the system.
- Authentication and Authorisation: Before each registered controller publishes a message, it needs to connect to the gateway’s MQTT Broker. A controller registers to the gateway and provides the device certificate, private key and gateway certificate, which are used for mutual authentication of the transport layer security (TLS) and the gateway. The gateway first verifies its identity before identifying whether it meets the requirements of the match policy to obtain a temporary token. If the controller’s credentials pass the authentication requirements and have the authority to obtain a temporary token, the gateway recognises the controller as a member of the local device and authorises it by sending a temporary token.
- User’s Request: After receiving the user’s control request from the controller, the gateway analyses the request—including which devices to control—the device settings, the device instructions and the changed device status values (e.g., fan volume, setting value of air conditioning and bulb brightness). Figure 4 shows the flowchart. After receiving the user’s request, the gateway first analyses the device to obtain the device status stored in the gateway. If the user wants to control the device in a controllable state (Algorithm 1), the gateway analyses the corresponding actions of the button or voice commands, for example, turning the button to adjust the strength of the device or recognising the voice stating ‘turn off’ to turn off the device. The gateway changes the state value of the device and analyses the related control instructions of the device, such as raw infrared data for turning the TV on and off, and *RESTful API* instructions to adjust the strength of the air conditioner. Finally, the gateway publishes the relevant instructions and device status information.
- Device Status: The gateway needs to record the status of all the devices on the local. This paper uses JSON files to store the status of the devices in the gateway. Each field has the relevant properties of the corresponding device. For example, electric fans have multiple attributes, including switch on or off, wind force, position, whether to swing and control type. For the gateway to process the logic control (Algorithm 2) when processing user requests, the JSON files store all the controlled device information in the system, such as the device type, current setting values, device status that the controller controls and the last received instruction, as shown in Figure 5. Then, the SDC obtains a copy file of the device status and displays information for the device status.

**Algorithm 1.** Device’s ControllableBegin: Device’s name1:controllable = the “control” field of device in JSONstate2:**if** controllable is True **then**3: return True4:
**else**
5: return False6:
**end if**
7:
**END**


Some environments have several controllers. For example, in the field of smart homes, each family member has one SDC, $SDC=\left\{SDC_{1},SDC_{2},\ldots ,SDC_{n}\right\}$, where n∈N. If $SDC_{1}$ controls a specific machine, $SDC_{1}$ must synchronise the set value changed by the home appliance to all SDCs in the field to prevent the local device from repeating operations. Whenever a user makes a device control request, the user controls the request message published to the gateway. After verifying its identity, the gateway updates the relevant device status in the original JSON file to show that $SDC_{1}$ is controlling the system. The gateway then publishes the device status and the device instruction to $\forall i\in N:SDC_{i}$. When $SDC_{i}$ receives the device status of the gateway, it updates the device status copy on the $SDC_{i}$ and displays it to the user. If $SDC_{i,i\neq 1}$ wants to control the same device as $SDC_{1}$, it needs to go through the logic control of the gateway and refuse the $SDC_{i,i\neq 1}$ request according to the timestamp. The centralised management of the JSON files in the gateway means that when there is a control request, a status copy is obtained, thus preventing duplicate or simultaneous operations.
**Algorithm 2.** Logic Control of GatewayBegin: JSON context of user request1:*msg* is **JSON context of user request**2:Get *SDCname*, *SDCstate*, *SDCstate_Topic*, and *SDCcmd_Topic* from **JSON device state**3:Get *device_name* and *device_state* from **JSON device state**4:**if** Controllable(*device_name*, *device_state*) is False **then**5: publish_message(‘False’, *SDCstate_Topic*) 6: return7:**end if**8:set_device_state(*SDCname*, *device_name*)9:Get device’s *type* from **JSON device state**10:**if** msg == ‘switch controlled device’ **then**11: Update the device that SDC is controlling in **JSON device state**12:**elif** msg == ‘control device’13: According user request set *device_set_value* and generate *command*
14: Using *device_name* and *device_set_value* to update device’s state15: Get *device_state* after update16: **if** device’s *type* is ‘smart device’ **then**17:  publish_message(*command* and *type*, *SDCcmd_Topic*)18: **elif** device’s *type* is ‘traditional device’19:  Get *TDCname*, *TDCstate*, *TDCstate_Topic*, and *TDCcmd_Topic* from **JSON device state**20:  publish_message(*command* and *type*, *TDCcmd_Topic*)21: **end if**22:**end if**23:publish_message(*device_state*, *SDCstate_Topic*)24:**END**

### 3.2. Smart Device Module

**Figure 6:**
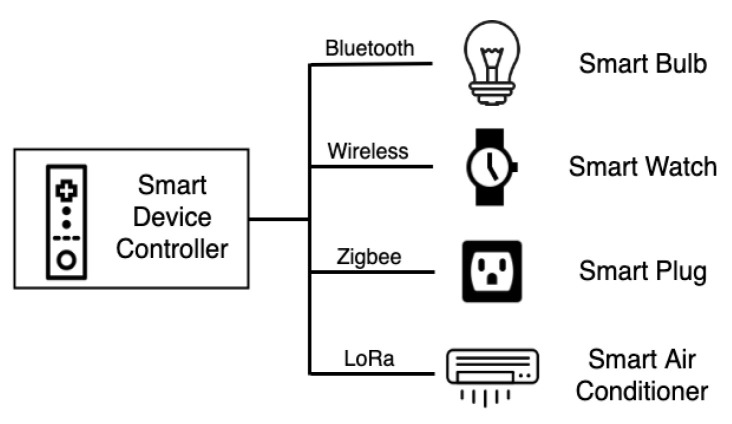
Wireless communication technology type of smart end device.

The SDC of the framework mainly controls smart devices [29] in the environment that use wireless communication methods such as Bluetooth, Zigbee, Wireless network and Radio Frequency Identification (RFID) (Figure 6). The SDC can be used to control the local device using various buttons, knobs and/or microphones. If necessary, an LCD screen or speaker is installed to indicate which device the controller is controlling and the device status value. When operating the SDC, the user needs to switch the SDC to the device to be controlled by pressing the button, turning a rotary knob or using voice control to publish the user’s request message to the gateway. The device logic, controlled by the SDC, determines the instruction corresponding to the request.

The X.509 certificate obtained by the SDC during the registration phase is used for gateway authentication. When connecting to the gateway, the gateway verifies the identity of the SDC and authorises the SDC to publish and receive relevant messages and obtain tokens issued by the gateway.

**Figure 7:**
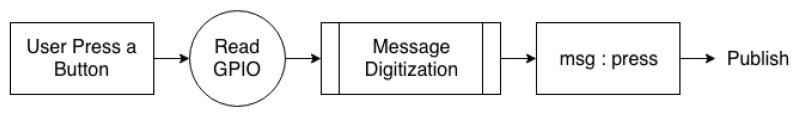
User’s action digitisation.

When a user uses the SDC to generate a user request, the action is digitalised and published. Figure 7 shows the process flow. The user presses a button on the controller to generate a ‘press’ message and a message is published. When the gateway obtains the message, it makes a logic judgment to generate an operation instruction and updates the JSON file for the device status. The device status copy and operation instructions are published to the controller, including the SDC and TDC (responsible for controlling traditional home appliances), and the controller completes the device control according to the instructions.

### 3.3. Voice Module

Voice control and reminders are effective in many areas of life, such as guidance for visually impaired [22] or handicapped [23] users. This type of system improves the convenience of system operation [33,34]. The ‘Echo’ [35] developed by Amazon is the most popular voice control product on the market today.

**Figure 8:**
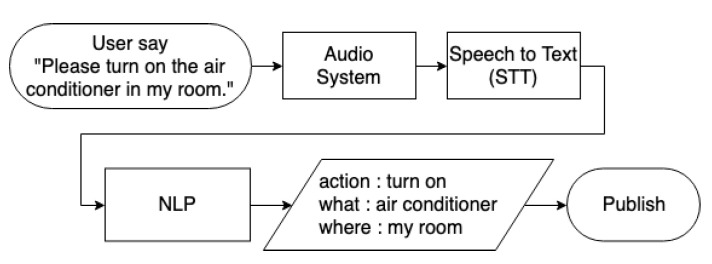
The flowchart of user’s voice semantic analysis.

The voice function analyses the intent expressed by the user’s voice through natural language analysis. This paper uses the voice module Amazon Lex [36] for natural language processing, which connects to cloud services via the internet. Lex is converted into words or symbols to analyse the intent, as shown in Figure 8. In the audio system, *SoX* [37] is used to read and write audio files in PI and acts as an audio player or a multi-track audio recorder. Then, the voice module listens for audio input, detects sound and starts streaming the audio to Lex. After semantic analysis, the user’s intention is published to the gateway for logic control processing. The gateway analyses the instructions and publishes them to the controller. Using voice commands can make the device seem more humane to users and is more helpful to some users, such as visually impaired people and people with reduced mobility. The voice module can also be combined with the SDC module, which combines manual operation and voice operation so that the controller can more directly express the device that the user wants to control through voice control, thus solving some user problems and making the system more intelligent.

### 3.4. IR-Emitter Module

Some environments use traditional devices that cannot connect to the internet, which is one reason why some industries have not yet made the digital transformation. It is costly to replace traditional devices with smart devices that have network functionality. To make a smooth digital transformation without incurring huge costs, the infrared controller module can be used to control traditional devices, such as old TVs, air conditioners, stoves and DVD players, using infrared control.

In this study, a single-chip development board is used for forwarding the control instructions, and an IR-emitter module is fixed in front of the infrared receiver of the device to be controlled, corresponding to a traditional device. After receiving the instructions published by the gateway, the IR-emitter sends infrared raw data corresponding to the functions of the traditional device to control the traditional infrared device.

### 3.5. Relay Controller Module

As mentioned in Section 1, some traditional devices cannot connect to the internet and need to be controlled by electric currents, such as electric fans, bulbs, dehumidifiers and even robot arms in factories. To remotely control this type of device, this paper uses a single-chip development board to forward the control instructions.

We therefore install a relay module in the single-chip development board, which uses a smaller electric current to control the larger electric current required by the traditional device. The internal solenoid of the relay controls the current loop to form a path and create a current switch. The module is installed on the socket of the control device. After receiving the operation instruction transmitted by the gateway, the relay controller makes or breaks the current to a path according to the operation instruction and controls the switching and strength of the electrical current to the device.

## 4. Experimental Results

This paper designs a prototype to test the feasibility of the framework, which uses a Raspberry Pi 3 and two Zero W. Raspberry Pi 3 represents the gateway, and Raspberry Pi and Zero W represent SDC and TDC, respectively. Then, as per the system architecture depicted in Figure 2, each module can obtain cloud services from the external network through the gateway. For the IoT scenario in this article, it is necessary to ensure that the local device can operate in a weak-signal or no-signal environment. We therefore conducted experiments when the gateway could not connect to the external network and observed the response time of each device to publish and subscribe after logic control. The response times were obtained for (1) the time to publish/subscribe between the same devices, (2) the devices that received the published messages between different devices, and (3) the logic control through the gateway.

### 4.1. Response Time of Publish/Subscribe Message between Same Device

This experiment was performed to obtain the response time of the publish/subscribe messages by the publishing end, receiving end and gateway when using the same device offline, respectively, with Pi 3 and Zero W. For the same device, messages are passed between different modular functions to measure the response time between publish/receive messages in an internet-less environment. As the publishing end, receiving end and gateway are all part of the same device, this method should be suitable for small environments, such as a room of a smart hotel or laboratory.

**Figure 9:**
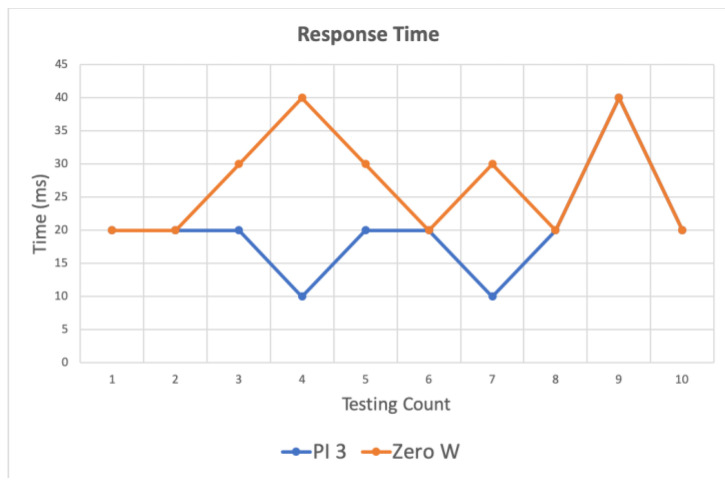
Response time of publish/subscribe for PI 3 and Zero W. The blue line shows that PI 3 is both a publisher and a subscriber. The orange line shows that Zero W is both a publisher and a subscriber.

**Figure 10:**
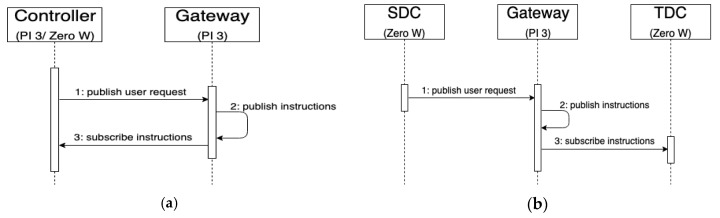
Time sequence diagram of publish/subscribe between (**a**) same devices and (**b**) different devices.

Figure 9 shows the response time between publishing a user request and receiving a control instruction at the gateway. Figure 10a shows the time sequence. As soon as the controller starts to publish the user requests, the gateway immediately publishes the feedback message after receiving the message published by the controller.

### 4.2. Response Time of Publish/Subscribe Message between Different Devices

**Figure 11:**
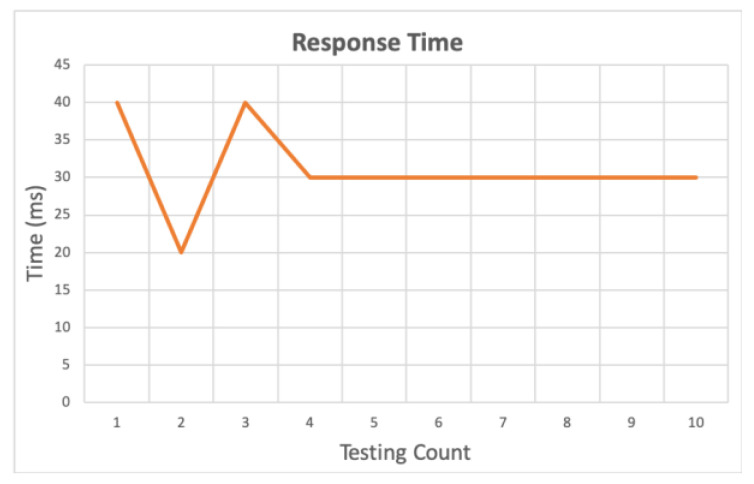
Response time of publish/subscribe between different devices. Pi 3 publishes a message as a publisher; then, Zero W subscribes a message as a subscriber.

This experiment uses Pi 3 as the gateway, one Zero W as the SDC and the other Zero W as the TDC. We measured the response time of different devices for publishing and receiving messages. In some scenarios, the SDC is used to publish user requests and operate smart devices and traditional devices, and the TDC forwards the signals. Figure 10b shows the timing sequence. After the SDC publishes the user’s request, it is sent to the TDC immediately after passing the gateway. Finally, the TDC forwards the instruction to the infrared raw date or relay switch to operate the traditional device. Figure 11 shows the results of the reaction time for the same experiment without any logic control.

### 4.3. Logical Control through Gateway

**Figure 12:**
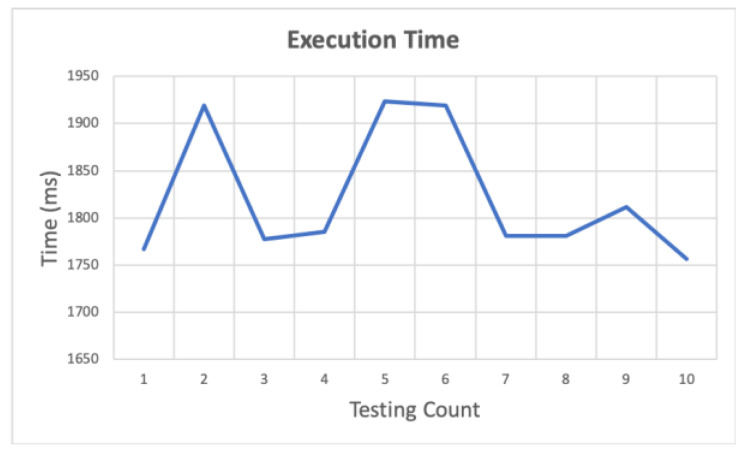
Execution time of Algorithm 2 by Python on PI 3.

In this experiment, the sender publishes the instruction message after the logic control of the gateway, and the SDC receives the message. The purpose is to analyse the response time, which is controlled by the logic control. In the experiment, the logic control of Algorithm 2 was used by Python, and Pi 3 was used as the gateway. After receiving the General Purpose Input/Output (GPIO) message, the algorithm reads the status of the device to be controlled and determines the button function corresponding to the GPIO. In this experiment, the button was pressed 10 times. The corresponding button functions are pressed to turn the device on or off. After the message is published, the gateway performs the logic control of the device to generate the device instructions. Finally, the instruction message is published. Figure 12 shows the execution time, which is an average of 1822.298 ms.

**Figure 13:**
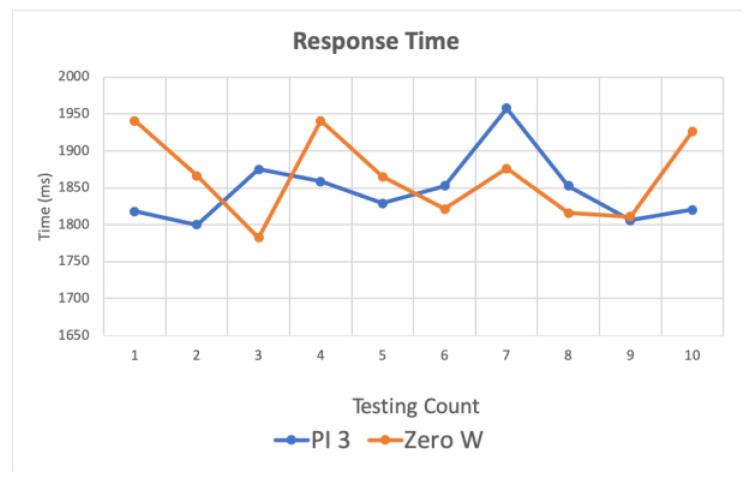
Response time for PI 3 and Zero W after logical control of gateway. The blue line shows that PI is both a publisher and a subscriber. The orange line shows that Zero W is both a publisher and a subscriber.

**Figure 14:**
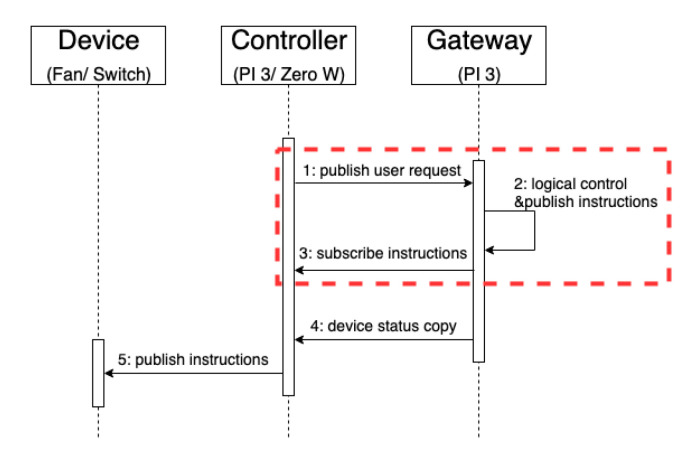
Timing sequence diagram of publish/subscribe at the same devices by logical control. This process simulated the smart-device controller (SDC) to control a smart device.

Figure 13 shows the relevant logic control instructions through the gateway. In the first experiment (1), the publishing end, receiving end and gateway are all the same device, Pi 3 and two Zero W, respectively. Figure 14 shows the timing sequence. The red frame in the figure is the execution time of Figure 13. First, the controller publishes the user’s request. After receiving the message published by the controller, the gateway generates device instructions through logic control. Then, the instructions are published to the SDC.

**Figure 15:**
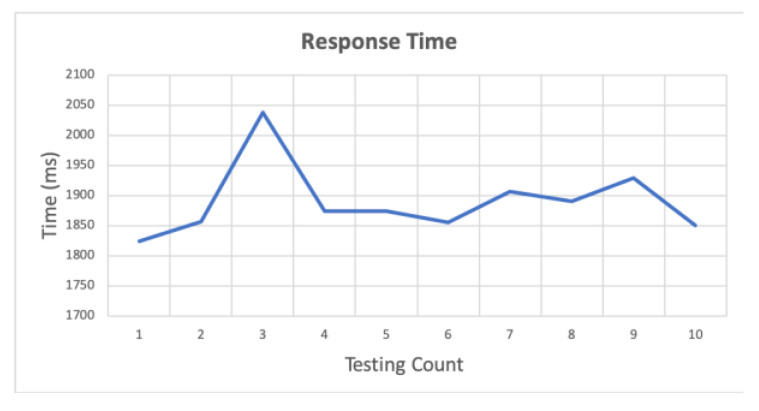
Response time of publish/subscribe between different devices after logical control of gateway. A Zero W as the SDC publishes a message, and then the other Zero W as the TDC subscribes a message.

**Figure 16:**
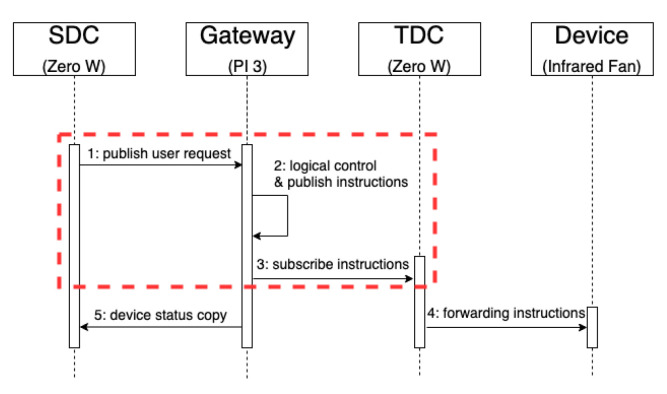
Timing sequence diagram of publish/subscribe between different devices by logical control. This process is to simulate the SDC to control a traditional device.

Finally, we use Pi 3 as the gateway, one Zero W as the SDC and the other Zero W as the TDC. Figure 15 shows the logic control-related instructions through the gateway. In the second experiment (2), the SDC publishes the user requests and operates both smart devices and traditional devices, and the TDC forwards the signals. In this experiment, the infrared fan is turned on or off by continuous pressing of the button 10 times. Figure 16 shows the complete timing sequence. The message is published to the gateway after the user presses the button. Then, the device instructions are generated after the logic control is performed by the gateway. Finally, the control instruction is published to the TDC, which controls the traditional device, and the device status copy is published to the SDC device information for the user. Figure 16 shows the measured response time in the red frame.

## 5. Use Case

The IoT has produced revolutionary changes by manufacturing sensors, generating and transmitting data, and combining mobile phones, tablets, clouds and major platforms to produce a variety of different applications ranging from smart homes to smart healthcare. The framework proposed in this research offers advantages for the application of IoT to various use scenarios, including smart warehouses, smart agriculture, smart homes, smart restaurants, smart medical care and smart factories. This section introduces how to choose modular functions and deploy the framework in smart hotels and smart warehouses.

### 5.1. Smart Hotel

With the changing times, many hotels are slowly transforming into smart hotels. However, because of the cost, it is not feasible to replace all existing equipment with smart devices. The modular framework proposed herein can provide a low-cost transformation for hotels, enabling them to provide intelligent services without needing to replace their existing devices.

Hotel rooms contain several operable types of equipment, such as heating and cooling systems, kettles, televisions, stereos, electric lights, smart grids and smart sockets. To apply a modular framework, the devices are divided into traditional devices and smart devices. Traditional infrared devices are controlled by an IR-emitter, while traditional mechanical devices are controlled by relays and use PWD or PID to control the strength. Then, the smart device is controlled by the SDC.

**Figure 17:**
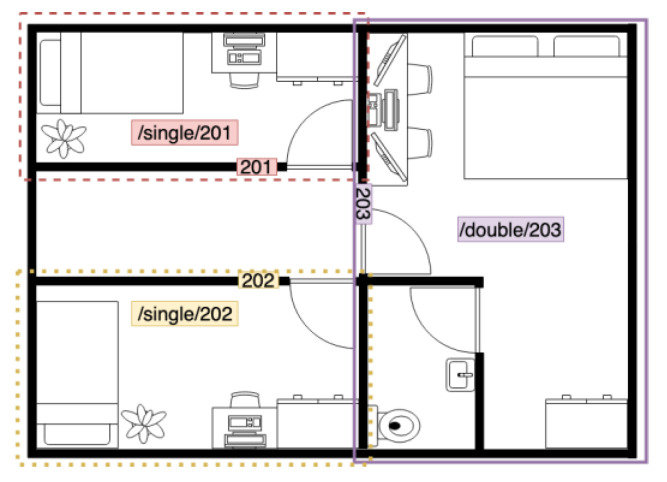
The example of room topic in smart hotel.

Note that as hotel rooms operate independently, each room requires an IoT modular framework, and each SDC or TDC receives MQTT operation messages. If each room is not distinguished, when the tenant operates their room equipment, the controller receives the instruction and controls the room equipment of another or all other rooms. Tantitharanukul et al. [38] claimed that the topic is named based on three attributes: ‘Objective’, ‘Location’ and ‘Owner’. To distinguish the topic of each room, room type and room number are used for determination based on Objective: *RoomType/RoomNumber*. The last instruction received in each room needs to be published to a different MQTT topic to solve this problem that distinguishes the topic of each room, so that each room has its MQTT topic, as shown in Figure 17. In Figure 17, each controller in the room corresponds to the room number, and each room has its topic, which uses the room number as a unique identifier. The gateway can obtain the room topic to which the controller belongs from the device state. With this method, even if these devices belong to the same gateway module, each controller can control a specific room.

### 5.2. Smart Factory

The modular framework proposed in this paper is suitable for various environments, including a smart warehouse, which contains all kinds of machines such as robotic arms, unmanned aircraft, unmanned vehicles, take-off and landing machines and robots [2,6,7,39,40]. Such a large environment typically comprises large shelves, shelters, high places and corners that can cause instability for wireless signals. If an IoT device exists in a space that cannot fully receive instructions, it could cause the device to fail to operate, resulting in damage to the device or human casualties.

It is thus necessary to ensure that the system can operate offline to ensure the reliability of the IoT system in a smart warehouse. An MQTT broker is installed on the gateway so that all data can be transmitted to the gateway to make logic judgments and generate operation instructions. Signal reliability can also ensure system reliability. The devices that need to be controlled in the warehouse must be classified. These devices, which are controlled by relay device controllers, include traditional devices such as take-off and landing machines, robot arms and stackers. By contrast, connected devices, such as drones or unmanned vehicles, are controlled by the SDC.

Typically, multiple workers control the devices in the warehouse, and these workers can use the SDC. Therefore, attention must be paid to the synchronisation between the devices. For example, if Worker A is using a lifter to pick up goods, the status of the lifter needs to be synchronised to the SDC of each worker; without this synchronisation, Worker B might be unaware that anyone is using the lift and directly operate the landing gear to pick up the goods on another shelf, thus causing an accident involving Worker A. Therefore, it is essential to obtain the device status in the warehouse from the gateway. If the device is determined to be in use, the gateway refuses access control to the controller.

## 6. Conclusions

This paper presents a framework that ensures the reliability of IoT system services so that even if the IoT system cannot connect to the network, the system can provide the services by edge computing. An edge device can not only transmit data but also preprocess data and analyse real-time data, which can support edge computing and provide logic control services. The gateway stores the related status of all devices in the group. When a user requests generation, it will perform edge computing on the device status to generate related instructions. The framework proposed in this paper can effectively reduce the difficulty of software deployment and maintenance and prevent unauthorised devices from performing masquerading attacks to ensure minimal system security and resilience. This article provided examples of how to deploy the system; the framework can be used to deploy an IoT system architecture suitable for various industries.

The experiments tested the operations of smart and traditional devices. During the system initialisation, the controller connects to the gateway and performs X.509 certificate authentication and authorisation to ensure that the system is not fraudulent. Once authorisation is successful, the system can operate.

In the experiment, this paper uses Pi 3 as the gateway to perform most of the computing functions; however, in the experiments in Section 4, using Pi 3 to make logic judgments is also somewhat time-consuming. Most of that time is spent on the logic control of the device functions. The more the device functions, the stricter the logic control needs to be and the more time needs to be spent. Scenarios requiring faster requirements or large fields, such as smart homes, should use a high operand mini-computer instead.

At this stage, traditional devices have been connected to the TDC. In future work, multiple environmental sensors, such as temperature and humidity, soil humidity, PM2.5 and human infrared sensors will be added. These environmental sensing data will be transmitted to the gateway and then to the server for machine learning, where the environmental data will be analysed before returning the recommended machine operations. Combined with indoor positioning to sense the user’s position, this system will provide complete and better smart control.
